# 552. Effect of *Lactococcus lactis strain plasma* (LC- Plasma) on immune response and symptoms in patients with mild COVID-19. Result of the multicenter, double- blinded, randomized controlled trial (PLATEAU study)

**DOI:** 10.1093/ofid/ofad500.621

**Published:** 2023-11-27

**Authors:** Kazuko Yamamoto, Tsuyoshi Inoue, Kenta Jounai, Ryohei Tsuji, Daisuke Fujiwara, Naoki Iwanaga, Takaya Ikeda, Toyomitsu Sawai, Yosuke Nagayoshi, Koji Hashiguchi, Yohji Futsuki, Yuichi Matsubara, Naoki Hosogaya, Katsunori Yanagihara, Koichi Izumikawa, Hiroshi Mukae

**Affiliations:** University of the Ryukyus Graduate School of Medicine, Nishihara, Okinawa, Japan; Nagasaki University Graduate School of Biomedical Sciences, Nagasaki, Nagasaki, Japan; Kirin Holdings Co Ltd, Yokohama, Kanagawa, Japan; Kirin Holdings Co Ltd, Yokohama, Kanagawa, Japan; Kirin Holdings Co Ltd, Yokohama, Kanagawa, Japan; Nagasaki University Hospital, Nagasaki, Nagasaki, Japan; Nagasaki Tokushukai Hospital, Nagasaki, Nagasaki, Japan; Nagasaki Harbor Medical Center, Nagasaki, Nagasaki, Japan; Kouseikai Hoapital, Nagasaki, Nagasaki, Japan; Japanese Red Cross Nagasaki Genbaku Hospital, Nagasaki, Nagasaki, Japan; Saiseikai Nagasaki Hospital, Nagasaki, Nagasaki, Japan; Juko Memorial Nagasaki Hospital, Nagasaki, Nagasaki, Japan; Nagasaki University, Nagasaki, Nagasaki, Japan; Nagasaki University, Nagasaki, Nagasaki, Japan; Nagasaki University, Nagasaki, Nagasaki, Japan; Nagasaki University, Nagasaki, Nagasaki, Japan

## Abstract

**Background:**

The COVID-19 epidemic has been repeated worldwide; however, easily accessible treatment options for patients with mild COVID-19 remain limited. Since the *Lactococcus lactis strain plasma* (LC- Plasma) enhances both the innate and acquired immune systems through the activation of plasmacytoid dendritic cells (pDCs), we hypothesized that the oral intake of LC-Plasma could ease symptoms in patients with mild COVID-19.

**Methods:**

The study protocol was registered in Japan Registry of Clinical Trials (jRCTs071210097). A total of 100 patients with mild COVID-19 were enrolled from January through March 2022 during epidemic timing of omicron BA.1 strain. Patients were randomly assigned by allocation factors of age, anti-viral medication, and SARS-CoV-2 vaccine status in a 1:1 ratio to LC-Plasma group (oral LC- Plasma- capsule, 200 mg/day, for 14 days) or placebo (oral placebo capsule, for 14 days). The primary endpoint was the change in total severity score of 8 subjective symptoms. Secondary endpoints include the change of each subjective symptom, SARS-CoV-2 viral loads, pDC activation, serum SARS-CoV-2 specific antibodies, type I interferons, and the proportion of subjects with emergency room visits or who were hospitalized.

**Results:**

A total of 50 patients in LC-Plasma group and 46 patients (4 omitted by declining the participation or unsatisfied criteria) in placebo group were analyzed. There was no difference between groups in patients’ background including age, sex, body mass index, and SARS-CoV-2 vaccination status (Table 1). The primary endpoint, total score of symptoms, was not different between groups (Figure 1A). As secondary endpoint, LC-Plasma group disappeared smell and taste disorders after day 9 compared to placebo group (p< 0.05, Figure 1B). Additionally, SARS-CoV-2 viral load was significantly decreased at day 4 in LC-Plasma group (p< 0.05, Figure 2A). Also, pDC decreased in the placebo group during the course, however, has maintained in LC-Plasma group (p< 0.05, Figure 2B). No patient was hospitalized or visited ER during the study. No adverse effect was reported except diarrhea observed in one patient in the LC Plasma group.

**Figure:**
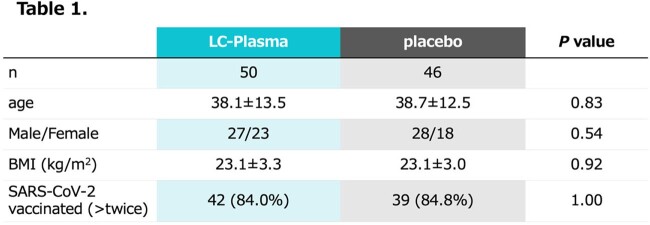

Patients' backgrounds

**Figure:**
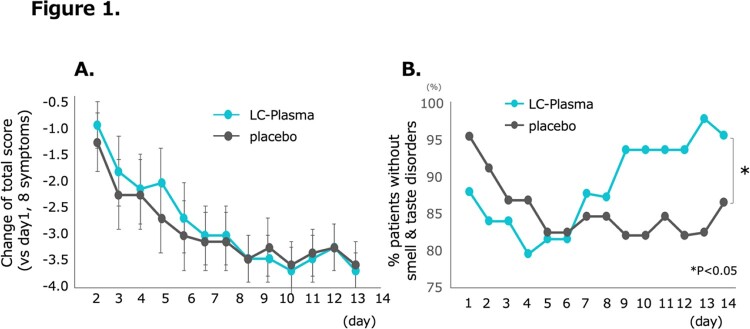

A. Change of total score (vs day1, 8 symptoms). B. % patients without smell & taste disorders

**Figure:**
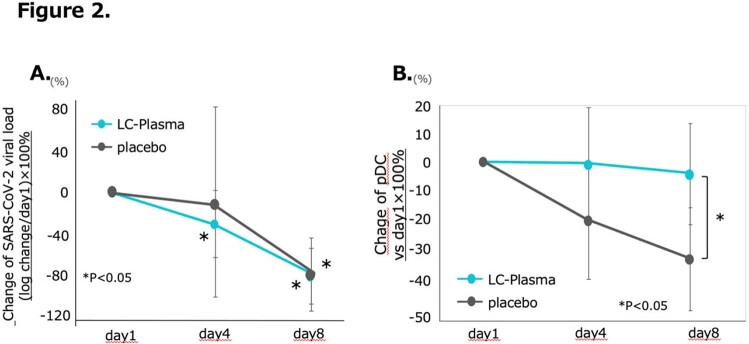

A. Change of SARS-CoV-2 viral load: (log change/day1)×100%. B. Chage of pDC (vs day1×100%)

**Conclusion:**

LC-Plasma is safe and a potential adjunctive treatment to maintain immunity, fasten viral eradication, and ease symptoms for mild COVID-19.

**Disclosures:**

**Kazuko Yamamoto, MD, PhD**, Fisher & Paykel Healthcare: Grant/Research Support|Kirin Holdings Co.: Grant/Research Support **Tsuyoshi Inoue, MD, PhD**, Kirin Holdings Co.: Grant/Research Support|Kyowa Kirin Co.: donated course **Kenta Jounai, PhD**, Kirin Holdings Co.: Employee **Ryohei Tsuji, PhD**, Kirin Holdings Co.: Employee **Daisuke Fujiwara, PhD**, Kirin Holdings Co.: Employee **Katsunori Yanagihara, MD, PhD**, FUJIFILM Toyama Chemical Co., Ltd.: Commissioned research|KYORIN Pharmaceutical Co.,Ltd.: Commissioned research **Koichi Izumikawa, M.D., Ph.D.**, Asahi Kasei Pharma Corporation: Grant/Research Support|Asahi Kasei Pharma Corporation: Honoraria|Astellas Pharma Inc.: Honoraria|DAIICHI SANKYO COMPANY, LIMITED: Grant/Research Support|DAIICHI SANKYO COMPANY, LIMITED: Honoraria|KYORIN Pharmaceutical Co.,Ltd.: Honoraria|Merck & Co., Inc.: Honoraria|Pfizer Japan Inc.: Honoraria|Shionogi & Co., Ltd.: Grant/Research Support|Shionogi & Co., Ltd.: Honoraria|Sumitomo Pharma Co., Ltd.: Grant/Research Support|Sumitomo Pharma Co., Ltd.: Honoraria|TAIHO PHARMACEUTICAL CO., LTD.: Grant/Research Support

